# The integrated stress response in cancer progression: a force for plasticity and resistance

**DOI:** 10.3389/fonc.2023.1206561

**Published:** 2023-08-03

**Authors:** Caleb L. Lines, Morgan J. McGrath, Tanis Dorwart, Crystal S. Conn

**Affiliations:** Department of Radiation Oncology, University of Pennsylvania, Perelman School of Medicine, Philadelphia, PA, United States

**Keywords:** cancer, stress response, mRNA translation, adaptation, drug resistance

## Abstract

During their quest for growth, adaptation, and survival, cancer cells create a favorable environment through the manipulation of normal cellular mechanisms. They increase anabolic processes, including protein synthesis, to facilitate uncontrolled proliferation and deplete the tumor microenvironment of resources. As a dynamic adaptation to the self-imposed oncogenic stress, cancer cells promptly hijack translational control to alter gene expression. Rewiring the cellular proteome shifts the phenotypic balance between growth and adaptation to promote therapeutic resistance and cancer cell survival. The integrated stress response (ISR) is a key translational program activated by oncogenic stress that is utilized to fine-tune protein synthesis and adjust to environmental barriers. Here, we focus on the role of ISR signaling for driving cancer progression. We highlight mechanisms of regulation for distinct mRNA translation downstream of the ISR, expand on oncogenic signaling utilizing the ISR in response to environmental stresses, and pinpoint the impact this has for cancer cell plasticity during resistance to therapy. There is an ongoing need for innovative drug targets in cancer treatment, and modulating ISR activity may provide a unique avenue for clinical benefit.

## 1 Introduction

Cancer therapeutics and diagnostics have expanded considerably over the past few decades, yet cellular adaptations persist, enabling resistance to chemotherapeutics, targeted therapies, and immunotherapies alike. This is partly due to the heterogeneity that arises within tumor populations, but limitations in widely utilized experimental approaches also hinder the identification of novel targets for the treatment of resistant tumors. The majority of clinical efforts focus on identifying therapies and biomarkers based on chromosomal alterations and RNA sequencing data which highlights global transcriptional changes. However, mRNA transcript abundance does not faithfully portray the phenotypical representation of gene expression as functional protein within a cell (1–3). This discrepancy partially emerges from the regulation of mRNA translation– the process by which mRNAs are selectively bound and deciphered to produce protein (4). Once thought of as a housekeeping process, protein synthesis is now known to be highly coordinated and subject to modulation to introduce additional layers of gene regulation that can also increase proteomic diversity (5, 6). Nearly 60% of our protein variations can be attributed to post-transcriptional processes, including RNA splicing, RNA epigenetics, and distinct utilization of 5’ and 3’ untranslated regions (UTR) along mRNA (1). Through these mechanisms and others, modulators of protein synthesis are now recognized as major contributing factors of altered gene expression and increased phenotypic diversity— including therapeutic resistance-enabling heterogeneity which arises between cancer cells of a single tumor.

The regulation of mRNA translation allows for rapid responses to pathological challenges that enable cells with dynamic survival advantages to swiftly alter their phenotype. This translational rewiring allows for cellular plasticity— the ability of cells to assume a range of diverse phenotypes in response to their environment (7). Cell state transitions are essential during development and for tissue regeneration, but cancer cells can abuse this plasticity to thrive in adverse conditions (whether in response to intrinsic oncogenic stresses or extrinsic clinical therapies) through non-transcriptional mechanisms (8). Understanding the ability of cancer cells to evade therapy through enhanced plastic tendencies for dormancy, stemness, or other advantageous states is quickly becoming a major focus for battling cancer progression. The key role in which translational regulation facilitates cancer plasticity is gaining interest as it may create a unique therapeutic opportunity (9).

One distinct translational program frequently activated in response to oncogenic events is the integrated stress response (ISR). In eukaryotes, the ISR has been implicated in a wide range of physiological events beyond cancer including metabolic reprogramming, memory formation, neurodegenerative diseases, and aspects of aging— emphasizing the pathway’s roles in various contexts (10–17). The ISR is an adaptive signaling response activated by a wide array of physiological or pathological stimuli in order to cope with stress and attempt to restore homeostasis (18). Cell intrinsic stressors can include depleted nutrients, low ATP, increased reactive oxygen species (ROS), and unfolded protein aggregates in the lumen of the endoplasmic reticulum (ER). Extrinsic triggers include hypoxia, further nutrient deprivation, DNA damaging agents, altered pH, and/or viral infection. Clinical therapies that create similar internal cell stress can also act as external triggers to induce the ISR. As such, the ISR is a highly conserved signaling cascade that is central for sensing these stressors. At the core of the ISR lies the phosphorylation of the eukaryotic initiation factor 2α (P-eIF2α), which is catalyzed independently by four unique serine/threonine kinases. In short, this single phosphorylation event downregulates global cap-dependent protein synthesis while selectively upregulating the translation of specific mRNA transcripts which function to restore cellular homeostasis. Severe stress and prolonged activation of the ISR can bypass homeostasis measures and activate separate factors to promote cell death. In this review, we discuss the mechanisms by which cancer cells hijack the ISR to rewire translation initiation in response to oncogenic stress and consider the potential for ISR-targeting therapeutics to combat cancer plasticity, progression, and treatment resistance.

## 2 Rewiring translation initiation by the ISR

**Figure 1:**
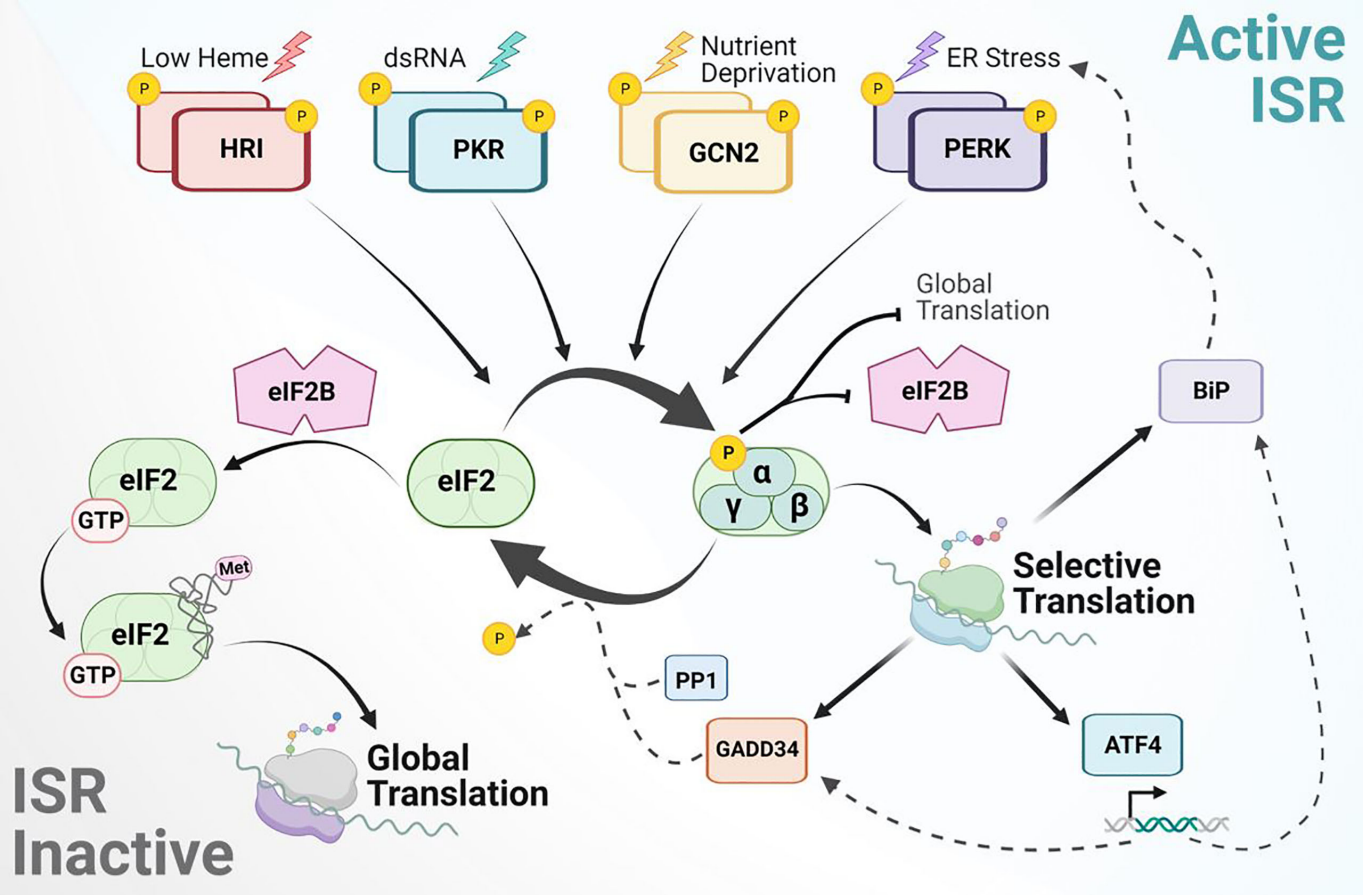
Mechanism of integrated stress response signaling to rewire protein synthesis. The ISR is activated by various stress stimuli recognized by four independent kinases to phosphorylate eIF2α. This blocks eIF2B GEF activity, leading to global attenuation of cap-dependent protein synthesis and activation of selective mRNA translation. Transcripts translationally upregulated during the ISR include BiP, ATF4, GADD34 and others not depicted. ATF4 is a main target that balances transcriptional regulation for adaptive response. ISR signaling is inactivated by stress-inducible phosphatases and their co-activators, such as PP1 with GADD34. Upon eIF2α dephosphorylation, eIF2B can bind eIF2 to catalyze GDP-GTP exchange and promote ternary complex formation for global protein synthesis.

When a cell is faced with antagonizing stress stimuli, an initial mitigation strategy is to conserve energy and resources by decreasing the rate of global mRNA translation. The ISR is a major regulatory network that initiates this drop in global translation through P-eIF2α while allowing for enhanced expression of select genes to promote cell adaptation (18). There are four kinases which phosphorylate eIF2α on serine 51, each activated by a specific set of cellular stresses through distinct regulatory domains (**Figure 1**). These domains activate the conserved kinase to promote trans-autophosphorylation. The ISR kinases include heme-regulated inhibitor (HRI), double-stranded RNA-dependent protein kinase (PKR), PKR-like ER kinase (PERK, also known as pancreatic ER kinase), and general control non-derepressible 2 (GCN2). HRI, a kinase predominantly expressed in erythroid cells, functions by adjusting the synthesis of globin to correspond with heme availability in response to oxidative stress (19, 20). It is similarly responsive to mitochondrial stress and contributes to clearing cytotoxic protein aggregates (21, 22). During viral infections, dsRNA activates PKR, downregulating the translation of virus-derived transcripts (23, 24). PKR activation has also been observed in response to ER stress, DNA damage, and uncharged mitochondria tRNAs (25). The kinase activity of PERK, an ER resident transmembrane protein, is induced through the unfolded protein response (UPR) and can be activated by oxidative stress from DNA damage, mitochondrial stress during times of nutrient deprivation, and hypoxia (26, 27). Under deprivation of charged tRNAs, GCN2 kinase activity is activated by two amino acid-sensing His-tRNA-like domains in its carboxy terminus functioning as cytoplasmic sensors of amino acid levels (28, 29). As such, a variety of nutrient deprivation conditions will activate GCN2 (30). GCN2 also binds the ribosomal complex P-stalk of the large ribosomal subunit and is activated during ribosome stalling, suggesting additional roles in monitoring ribosome stress (31, 32). In subsequent sections, we expand upon the specificity of these kinases with an emphasis on their identified roles in oncogenic signaling and how their adaptive responses can be targeted in support of cancer remission.

Upon phosphorylation of eIF2α at serine 51, the binding affinity to eukaryotic initiation factor 2B (eIF2B) increases (33, 34). eIF2B is a heterodecamer guanine nucleotide exchange factor (GEF) which binds to the γ-subunit of eIF2 to catalyze GDP-GTP exchange on the un-phosphorylated pool of eIF2-GDP (35, 36). When eIF2B is sequestered by P-eIF2α, P-eIF2α acts as a competitive inhibitor and prevents the activation of eIF2 required for global translation initiation. Canonical translation in eukaryotes typically initiates when a charged initiator methionine-transfer RNA (Met-tRNAi) recognizes an AUG start codon following delivery to the 40S ribosomal subunit by eIF2-GTP (37). The term “ternary complex” refers to this association of Met-tRNAi, eIF2, and GTP. The ternary complex assembles with the 40S ribosomal subunit and other eIF components to form the 43S preinitiation complex (PIC) (38). Recruitment of the 43S PIC onto mRNA by the cap binding complex (eIF4F: consisting of eIF4E, eIF4G, and eIF4A) initiates global protein synthesis and scanning of the mRNA 5’untranslated region (UTR). When formation of the PIC is uninterrupted and the complementary start codon sequence is recognized with Met-tRNAi, the 60S ribosomal subunit joins and releases select eIFs to promote translation elongation in addition to recruitment of other elongation factors. However, upon activation of the ISR, sequestering of eIF2B by P-eIF2α inhibits ternary complex formation (39). It is through this mechanism that the ISR causes a reduction in global protein synthesis (**Figure 1**).

The interactions between major ISR factors are highly complex and context specific. For example, the kinases and phosphatases involved in the phosphorylation and dephosphorylation of eIF2α, respectively, rely on specific higher order contacts for enzymatic activity to take place. This is due to eIF2α substrate residues establishing analogous contacts with both the phosphatases and kinases (40). Additionally, as noted above, eIF2B serves as a GEF for eIF2 enabling activation of the initiation factor. When eIF2α becomes phosphorylated, an S-loop in its protein structure becomes altered, subsequently transforming the factor into a high-affinity inhibitor of eIF2B by sequestering the catalytic domain (41). A small molecule inhibitor of the ISR, ISRIB, can reverse the effect that P-eIF2α has on global translation not by altering P-eIF2α itself, but by binding to and enabling higher-order assembly of the decameric eIF2B holoenzyme. This enhances eIF2B’s stability and enzymatic activity to overcome the ISR and restore global translation (42–44). Targeting eIF2 interactions has promoted a better comprehension of the ISR function and role in altering mRNA translation between states of growth and adaptation with promise for potential therapeutics.

While the ISR is associated with downregulation of global-canonical translation, numerous transcripts are preferentially translated for cell survival in adverse conditions. A variety of *cis*-regulatory domains have been found to contribute to this translational selectivity, including upstream open reading frames (uORFs), internal ribosome entry sites (IRES), RNA tertiary structures, regulatory protein binding sites, and epitranscriptomic modifications (45, 46). The most notable mechanism utilized during the ISR is regulation through uORFs. An uORF is a translatable sequence with its initiation codon upstream of the main ORF (mORF). Under normal conditions, the presence of uORFs within the 5’ UTR of an mRNA transcript antagonize translation of its mORF (47, 48). This reduction occurs due to the 5’-3’ scanning involved in cap-dependent translation. Once the PIC reaches an initiation codon, the eIF2-bound GTP is hydrolyzed, and the 60S ribosomal subunit joins. This displaces the initiation factors and forms the complete ribosome, prompting translation to begin. However, induced phosphorylation of eIF2α— and the resulting reduction in the ternary complex— can overcome these inhibitory effects and allow reinitiation at the mORF. The most well-known translational target downstream of ISR signaling is the activating transcription factor 4 (ATF4), which contains two uORFs; the second uORF overlaps with the start of the protein coding sequence in the mORF (49). The first uORF promotes ribosome scanning and re-initiation of the ribosome downstream at the second uORF whose translation prevents synthesis at the mORF. Upon stress, ISR activation slows the turnover of the ternary complex allowing the 40S subunit to scan through the second uORF to reinitiate at the start codon of ATF4’s mORF (50). This translationally controlled increase in ATF4 expression *via* the ISR leads to downstream transcription of pro-survival genes to respond to cellular stress (30, 51, 52). Interestingly, ATF4 promotes transcription of the Growth Arrest and DNA-Damage-Inducible 34 protein (GADD34). GADD34 is the regulatory factor of eIF2α’s protein phosphatase (PP1) and is also regulated through selective translation of uORFs (53, 54). Together, GADD34:PP1 deactivate the ISR by dephosphorylating eIF2α and promote a return of global protein synthesis (**Figure 1**).

Notably, one of the striking ways in which the ISR rewires initiation is through the utilization of non-canonical translation start sites (55, 56). Translational tracing in T-cells showed that during ISR activation the translation of BiP, a molecular chaperone of the HSP70 family, was sustained using uORFs in the 5’UTR of BiP mRNA. Translation at the uORFs was not initiated by the canonical AUG start codon, but rather upstream at UUG or CUG initiation sites, requiring the assistance of eIF2A (45). eIF2A binds to non-canonical initiator tRNAs and delivers them to the small ribosome subunit without requiring the same GTPase activity necessary for eIF2 function. This ultimately aids in initiation during stress conditions when eIF2 is inhibited. eIF2A functions synergistically with eIF5B— the latter of which provides ribosome-binding and GTPase functions (57). The usage of uORFs for alternate translation initiation during ISR activation is also subject to regulation *via* mRNA modifications, most notably N^6^-methyladenosine (m^6^A) methylation. m^6^A is asymmetrically distributed in mammalian mRNA transcripts. Under heat shock, however, m^6^A is preferentially deposited within the 5’ UTR of nascent transcripts (58). This increased methylation can modulate start codon selection and promote cap-independent translation initiation of HSP70 (59). In response to GCN2 activation by nutrient deprivation, the demethylase ALKBH5 is recruited to ATF4 mRNA to erase m6A. This reduction of uORF methylation reduces translation of the uORF to enable selective translation of the ATF4 mORF (60). The methods by which the ISR can rewire translation initiation are diverse, and cancer cells ultimately use ISR signaling during tumor progression to overcome oncogenic and therapeutic stresses.

## 3 Oncogenic stress activating the ISR

The multistep acquisition of tumorigenic traits as cells undergo neoplastic transition is highly heterogeneous due to the continuous alteration of gene expression to balance the demands of growth and adaptation for survival. This varies significantly between cancer types/subtypes and is further dependent on tissue specificity and site of transition (61). The use of single-cell RNA sequencing analysis has clearly demonstrated vast tumor heterogeneity (62). Multiple sources contribute to these heterogenetic populations. Advantageous mutations, chromosomal alterations, and epigenetic modifications may rise to prevalence within a tumor cell subset, producing a population evolved to combat the specific intrinsic and extrinsic stresses of the microenvironment. Other contributors to tumor heterogeneity, operating at post-transcriptional and translational levels, are often overlooked. Together, these reversible adaptive mechanisms constitute cell plasticity. While adaptive mechanisms may give rise to evolutionary alterations, both can confer therapeutic resistance to tumors. Here we focus on the capacity of oncogenic stress to activate adaptive pathways. In order to prevent and counter treatment resistance, one must ask: what are the mechanisms by which individual tumor cells and broader tumor subpopulations acquire resistant phenotypes?

**Figure 2:**
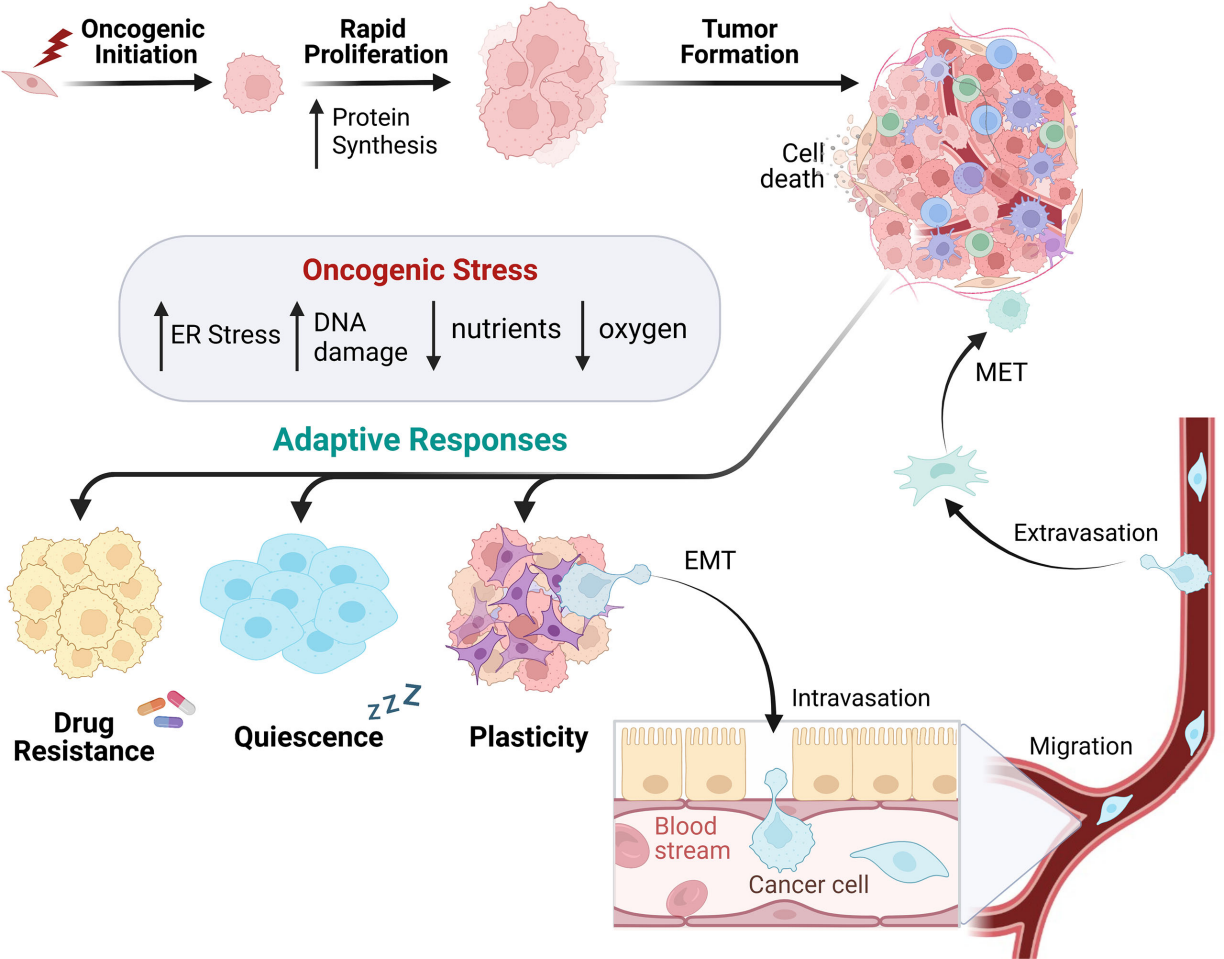
Cancer cell development utilizes heterogenetic adaption for tumor progression. In response to oncogenic lesions, protein synthesis is remodeled to enable rapid growth supporting tumor formation. Intracellular and extracellular oncogenic stressors subsequently alter the cancer cell phenotype, leading to the utilization of adaptive responses to promote survival. This can occur *via* drug resistance, cellular quiescence, and/or plasticity. Cells can promote a plastic phenotype by utilizing epithelial to mesenchymal transition (EMT) to migrate through the blood or lymphatic system and form metastases in other parts of the body.

One fundamental characteristic during cancer initiation is the deregulation of cell division, which eventually leads to uncontrolled cellular proliferation usually coupled to aberrant global protein synthesis (**Figure 2**) (63). Rapid oncogenic growth that occurs during tumor formation leads to a higher demand on the translational machinery (64, 65), resulting in a greater metabolic burden overall. These demands, in turn, place enhanced pressure on the proteostasis network (including ISR signaling) to balance nutrient demand for increased protein synthesis. This includes maintaining protein quality and proper peptide folding to prevent ER stress and activation of the UPR (66). Genomic instability is an enabling characteristic of cancer progression, and it synergizes with an increased metabolic rate and decreased oxygen availability. This leads to an increase in ROS due to increased oxidative and mitochondrial stress (67). These intrinsic tumorigenic pressures often result in a reconfiguration of the tumor microenvironment (TME). The rapid oncogenic growth during tumor formation also drains the local environment of nutrients and oxygen while limiting blood vessel perfusion to the core of the tumor, resulting in arid conditions (68). This challenges cancer cells to ration their resources, often perturbing metabolic usage towards aerobic glycolysis leading to decreased pH in the TME (69). Combined, these continuous stresses should disrupt the ability of the cell to regain homeostasis; however, activating adaptive pathways to promote dynamic cell state changes can promote survival. Active ISR results in lower global translation, decreased nutrient waste, enhanced pro-homeostasis factors, and improved survival. ISR signaling for adaptive mRNA translation can enable cancer cells to not only survive, but to enhance proliferation and progression within a stress-filled TME. The stress signals may vary by tumor type and location, but these adaptations are crucial for oncogenic survival.

The ISR is often exploited during oncogenesis, from cancer initiation to advanced metastasis and evasion of immunosurveillance. One seminal paper in the field identified that translational rewiring through ISR signaling, originating at pre-neoplastic stages, is a central player in skin cancer (70). Utilizing ribosome sequencing alongside an RNA interference-based screen, they observed that global mRNA translation was surprisingly lower than in normal cells due to ISR signaling for selective translation through uORFs requiring eIF2A for tumor initiation and progression. The ISR is also activated in pre-adenocarcinomas downstream of two major oncogenic lesions that promote metastatic-lethal prostate cancer (PTEN loss with MYC amplification) (71). In this context, eIF2α is phosphorylated in early neoplasia and signaling through PERK is required for tumor development. Inhibiting the ISR with ISRIB directly restores global protein synthesis, causing tumor regression and cell death in aggressive prostate cancer within genetic murine models and patient derived xenografts. The direct mechanism of activation is still not fully understood in these contexts, but PERK signaling is often observed for initiation and tumor progression due to oxidative stress, ER stress, and DNA damage (72–74). In lung adenocarcinoma, increased PERK activation likewise correlates with regions of higher proliferation, invasiveness, and tumor growth in patients. Specifically, P-eIF2α results in translational repression of the phosphatase DUSP6, promoting KRAS tumorigenesis and in turn a worse prognosis. ISRIB inhibits this translational repression and promotes tumor regression (75).

GCN2 has also been identified to promote prostate cancer by maintaining nutrient homeostasis (76). Intracellular amino acid levels are disrupted by GCN2 inhibition in metastatic prostate cancer cell lines, as GCN2 is necessary for transporter gene expression downstream of ATF4 (77). In the case of pancreatic cancer, cells relying on glycolytic pathways for energy have perpetually active ISR through GCN2-mediated ATF4 expression (78). Subsequently, ATF4 expression increases asparagine synthetase activity and the resulting asparagine is released into the TME. This asparagine can be taken up by cancer cells relying on cellular respiration and enable their proliferation even when respiration is blocked. In non-small cell lung cancer, KRAS also promotes asparagine biosynthesis in response to nutrient stress by regulating ATF4 transcription. However, ISR activity is still necessary to enhance ATF4 expression *via* translational regulation (79). The deletion of ATF4 alone, a single downstream ISR target, has been successful in significantly slowing MYC-driven tumor progression that relies on both GCN2 and PERK signaling in lymphomas (80).

The influence of the ISR is not solely confined to individual cancer cells. Rather, the ISR is used throughout the heterogeneous TME to enhance broader tumor resistance and persistence. In the context of melanoma and pancreatic tumors, a conditional knockout of ATF4 results in delayed tumor growth due to deficient vascularization (81). ATF4 was identified for regulating major amino acids of collagen, thus driving cancer-associated fibroblasts’ function for shaping the extracellular matrix to support tumor progression and metastasis. Independently, the TME cues ATF4 transcription of phosphoglycerate de-hydrogenase (PHGDH) in endothelial cells triggering altered metabolism towards glycolysis and aberrant over-sprouting vascularization in glioblastoma (82). This hostile vascularization presents a physical barrier to immune cells, hindering immunotherapies. ISR signaling also directly promotes non-canonical translation of programmed death ligand 1 (PD-L1) to escape immunosurveillance (83). Like ATF4, PD-L1 has an uORF inhibiting translation that is bypassed due to ISR. Utilizing an aggressive model of liver cancer, the authors showed that the non-canonical translation of PD-L1 is directly required for metastasis to the lung. The same immunosurveillance was observed in lung cancer, where a striking impairment of heme production resulted in the activation of HRI and enabled inhibitory uORFs of PD-L1 to be bypassed by the cancer cells’ translational machinery (84). Enhanced translation of PD-L1 decreases the ability of the local immune system to recognize and destroy the rapidly growing cancer. The increased presence of PD-L1 results in an overall suppression of T cell activity in the TME and prohibits T cell proliferation. The non-canonical translation necessary for the targeted translation of PD-L1 during ISR activation requires the activity of the alternative initiation factor, eIF5B. Understanding the broader role of eIF5B for ISR activity and immune regulation may present therapeutic opportunities to increase the susceptibility of immunologically ‘cold’ tumors (85).

## 4 ISR for resistance to cancer therapy

It is understood that cells are pre-programmed to differentiate to a particular cell type (“a cell fate”). However, cells which were once differentiated possess the ability to phenotypically change their characteristics as a means of survival (86). This plasticity allows cells to evade apoptosis and obtain favorable traits to aid in their progression. During tumor development, individual oncogenic lesions or adaptations can push a population of cells into cell cycle arrest, promoting quiescence and often drug resistance (**Figure 2**). Activation of ISR signaling is recognized for selectively up-regulating translational expression of a p21 transcript variant that contributes to cell cycle arrest and promotes cell survival (87). Recent work discovered that distinct nutrient deprivation in liver cancer induces cell-cycle arrest and quiescence through GCN2-mediated translation of p21, leading to therapy resistance (88). The amino acid transporter SLC7A1 was specifically required for arginine import and decreased with GCN2 inhibition, indicating that SLC7A1 may be a downstream component of the ISR. Inhibiting GCN2 in this arginine-deprived environment drove a senescent phenotype, restoring the hepatocellular carcinoma cells’ vulnerability to further treatments. Likewise, PERK signaling aids in the maintenance of quiescence in stem cells and is essential for the survival of progenitor cells (89, 90). The ability of cancer cells to transition phenotypes to promote tumor plasticity and survival often relies on the GCN2 and PERK pathways.

Cancer cells are also capable of transiting from an epithelial phenotype to a mesenchymal one. This phenomenon, termed epithelial-to-mesenchymal transition (EMT), is utilized in normal development and exploited by developing cancers to invade surrounding tissues and metastasize (**Figure 2**). Many carcinomas will separate from neighboring cells via loss of cadherin junctions and obtain mesenchymal characteristics to metastasize to secondary locations within the body. During this transition, cells may have a combination of both epithelial and mesenchymal characteristics, existing in a “partial-EMT” state where their cellular structure is plastic. This plasticity facilitates migration and survival (91). Several EMT transcription factors are capable of silencing epithelial gene expression and promoting mesenchymal gene transcription. Examples include SNAI1, ZEB1, and TWIST1, all of which have been shown to drive this transition (92). Characterizing this process has proven difficult due to the cells’ plasticity and capability to revert to an epithelial state once they have reached their new metastatic site *via* MET (mesenchymal to epithelial transition). This reverse process of EMT allows cells to regain the ability to anchor within their new environment and restart their growth phase (**Figure 2**) (93). The EMT-MET spectrum allows cells to gain favorable traits under an array of environments, stressors, and functional demands. Upon arrival at a secondary site, cells need to regress back to an epithelial state, attach to the basement membrane, and begin the process of colonization.

The ISR can enable cancer cells to dynamically alter their cellular state depending on recognition of specific cues, whether at the cell-intrinsic or broader TME level. It has previously been stated that the UPR, another important cellular stress response that overlaps with the ISR through PERK phosphorylation of eIF2α, plays an important role in recognizing the intensity and duration of ER stress caused by an overburden of unfolded proteins. This pathway’s ability to identify stress stimuli is critical in determining cell fate, driving the cells towards either homeostatic activation of survival pathways or maladaptive activation of apoptosis (94). Similar to the UPR, the ISR has been increasingly implicated in modulating cell fate and cell state, leading to increased survival in the context of disease and providing mechanisms for resistance to cancer therapy (95). A hallmark of UPR and ISR activity, PERK signaling, is activated during EMT progression (96). PERK activation was identified for selective translation of genes which enables the cell to combat stress, transition toward a mesenchymal phenotype, and resist chemotherapy. Additionally, unique mRNA isoforms of EMT transcription factors (*SNAIL*, *NANOG*, and *NODAL*) were identified in breast cancer and are selectively translated under ISR activation through their 5’UTR (97). These transcriptional repressors are crucial drivers of EMT, implying that ISR signaling may be required for survival through cell plasticity.

ISR activation not only enhances the ability of tumors to successfully enter a mesenchymal state but also confers resistance to therapeutic interventions. Poorly differentiated tumors (portraying a mesenchymal phenotype) are better suited to tolerate chemotherapy, while well-differentiated tumors are more sensitive to treatment (86). Furthermore, the ISR has been directly linked to preventing chemotherapeutics from having their intended effect during cancer treatment. In pancreatic cancer, the ISR plays a critical role in providing resistance to gemcitabine— a common chemotherapy for pancreatic cancer. Gemcitabine treatment resulted in P-eIF2α, which leads to the downstream induction of ATF4. Successive inhibition of ATF4 expression in gemcitabine-treated cells enhances apoptosis (98). In BRAF-mutated melanoma, the presence of chronic ER stress leads to ISR activation by enhancing P-eIF2α expression and contributes to chemoresistance through a dysregulation of autophagy. When ER stress is inhibited using the induction of chemical chaperones, autophagic activity is reduced and apoptosis increases in the BRAF-mutated melanoma (99). Furthermore, it has been demonstrated that induction of the ISR in human gastric cancer cells provides extra chemotherapeutic protection from the apoptotic capabilities of cisplatin and requires the presence of the cystine/glutamate antiporter, xCT, for the development of cisplatin resistance (100). In tumor cells, utilization of ISR activity is able to induce plastic tendencies, ultimately preventing the effects of therapeutic treatments intended to inhibit oncogenic growth and induce cancer death. Paradoxically, the same ISR mechanisms that contribute to highly resistant tumor phenotypes can be either exploited or inhibited to provide therapeutic relief.

## 5 Targeting the ISR during cancer therapy

**Figure 3:**
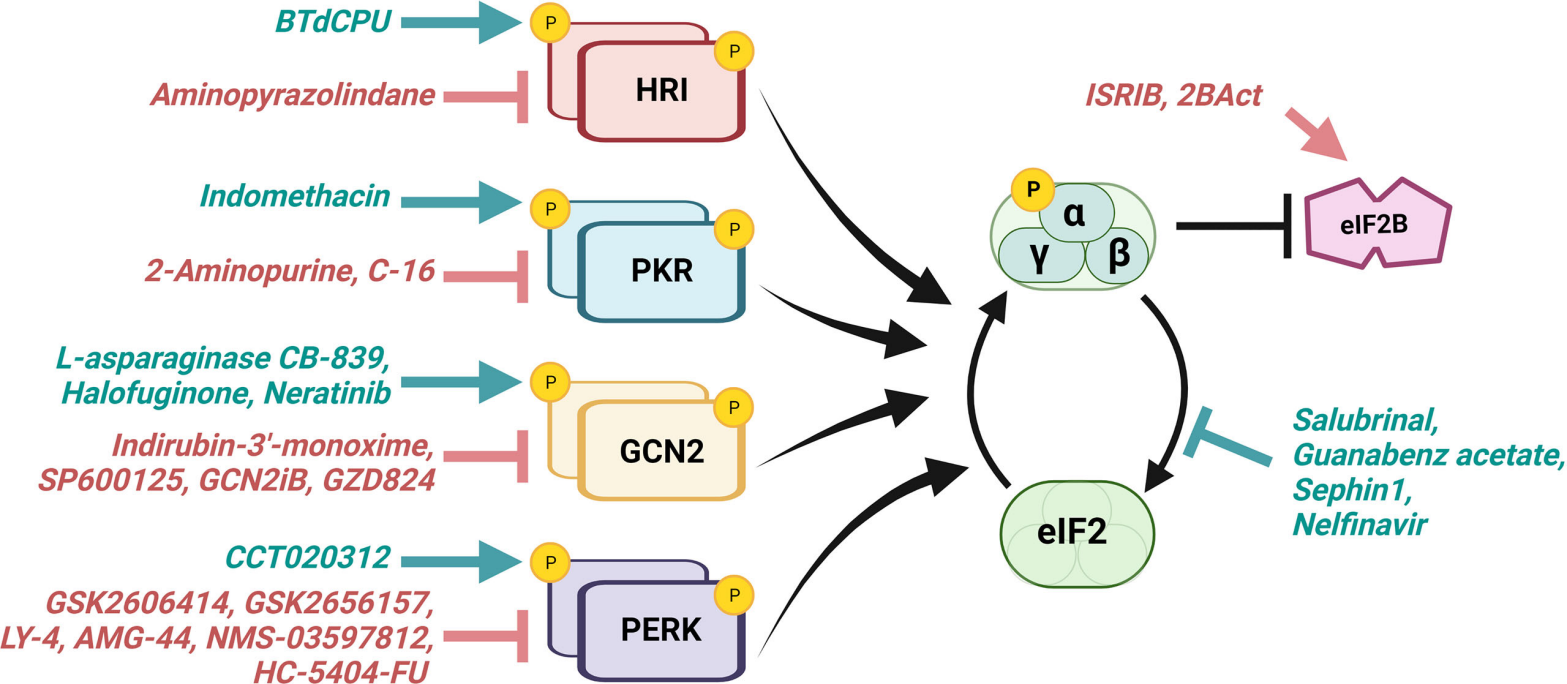
ISR-directed drugs target eIF2α kinases, eIF2α phosphatases, and eIF2B. Various therapeutics aim to activate (teal) or inhibit (red) the ISR for the treatment of cancer and other diseases. Those which promote the ISR aim to excessively activate a cell’s innate response mechanisms, driving that cell towards death, whereas those which inhibit the ISR aim to hinder the cell’s ability to adapt for survival under stress. eIF2α kinases serve as targets for both activators and inhibitors. Other current ISR-targeting drugs include eIF2α phosphatase inhibitors and eIF2B activators. Several of these therapeutics have shown promise in preclinical and/or clinical research described in **Table 1**.

Ongoing therapeutic efforts aim to manipulate cancer cells’ intrinsic adaptive mechanisms to combat tumor growth (101–103). While the ISR often functions to restore homeostasis by promoting adaptations during cancer progression for survival, robust and prolonged activation of ISR can drive cell death. It has been suggested that the eIF2α kinases which initiate ISR may also have a role in determining whether the response encourages adaptive survival or cell death, with PKR primarily noted as pro-apoptotic (104, 105). Because of its capacity to both maintain and shift the balance between cell survival and cell death mechanisms, the ISR has garnered significant attention as a potential target for modulation and exploitation of cellular adaptation (17, 95). Manipulating ISR activity in combination with other therapeutics may be useful in the treatment of cancer and is becoming an attractive target for pharmacologic intervention. Several drugs that activate or inhibit the ISR are being used actively in both primary research and clinical trials for cancer treatment (**Figure 3**; **Table 1**).

**Table 1:** ISR compounds in cancer treatment.

Effect on ISR	Target	Drug	Clinical Status (in cancer)	Cancer Types (not exhaustive)	References (not exhaustive)
**Activating**	HRI & PKR (via DRD2 inhibition)	ONC201	Phase II Phase II Phase III	Female reproductive cancers H3K27M-mutant glioma H3K27M-mutant glioma	NCT04055649 NCT05580562 NCT05476939
		ONC206	Phase I Phase I	CNS tumors CNS tumors	NCT04541082 NCT04732065
		ONC212	*Not Applicable (NA)*	• *in vitro*: various solid & hematological cancers • xenograft mouse models: Glioblastoma	Prabhu et al. 2020 (106)
	HRI	N,N'-diarylurea (BTdCPU)	*NA*	• *in vitro*: melanoma, breast cancer • xenograft mouse models: breast cancer	Chen et al. 2011 (107)
	PKR	Indomethacin (INDO)	Phase I, II Phase II	Prostate cancer Cervical cancer	NCT02935205 NCT03267680
	GCN2	L-asparaginase	FDA approved (multiple forms)	Acute lymphocytic leukemia	Maese and Rau 2022 (108)
		CB-839	Phase I Phase II	NSCLC Advanced NSCLC	NCT04250545 NCT03831932
		Halofuginone	Phase I Phase II	• xenograft mouse models: melanoma and esophageal cancer Advanced solid tumors, Sarcoma	Tsuchida et al. 2017 (109) NCT00027677 NCT00064142
		Neratinib	Phase I Phase II Phase III	• *in vitro*: Glioblastoma Advanced Malignant Solid Tumors, NSCLC, Breast Cancer	Tang et al. 2022 (110) NCT00768469 NCT01827267 NCT00878709
	PERK	CCT020312	*NA*	• *in vitro*: colorectal cancer, breast cancer	Li et al. 2020 (111) Lei et al. 2021 (112)
	eIF2α phosphatases	Salubrinal	*NA*	• *in vitro*: multiple myeloma, leukemia	Schewe and Aguirre-Ghiso 2009 (113); Drexler 2009 (114)
		Guanabenz acetate	*NA*	• *in vitro*: hepatocellular carcinoma, glioblastoma, breast cancer • xenograft mouse models: triple-negative breast cancer	Kang et al 2019 (115) Ho et al 2021 (116) Haggag et al 2021 (117)
		Sephin1	*NA*	• *in vitro*: anaplastic thyroid carcinoma • allograft and mouse models: melanoma and triple-negative breast cancer	Cao et al. 2019 (118) Wang et al. 2023 (119)
		Nelfinavir	Phase I Phase I, II Phase III	Vulvar cancer Solid tumors, Prostate cancer Cervical cancer	NCT04169763 NCT05036226 NCT03256916
**Inhibiting**	GCN2	Indirubin-3'-monoxime	*NA*	• *in vitro*: osteosarcoma, breast cancer, multiple myeloma • xenograft mouse models: multiple myeloma	Zhang et al. 2019 (120) Dilshara et al 2021 (121) Yu et al 2022 (122)
		SP600125	*NA*	• *in vitro*: various solid cancers • xenograft mouse models: bladder cancer	Cicenas et al 2017 (123) Yu et al 2019 (124)
		GCN2iB	*NA*	• *in vitro*: Acute lymphocytic leukemia • xenograft and mouse models: Hepatocellular carcinoma, prostate cancer	Heydt et al. 2021 (125) Cordova et al. 2022 (76) Missiaen et al. 2022 (88)
		GZD824	Phase I Phase II Phase II Phase III	Ph+ Acute lymphocytic leukemia Ph+ Acute lymphocytic leukemia Acute Leukemia Chronic Myeloid Leukemia	NCT05495035 NCT05521204 NCT05594784 NCT05311943
	PERK	GSK2606414	*NA*	• *in vitro*: various cell lines, Multiple Myeloma	Bagratuni et al. 2020 (126)
		GSK2656157	*NA*	• *in vitro*: various cell lines • xenograft mouse models: pancreatic cancer and multiple myeloma	Atkins et al. 2013 (127)
		LY-4	*NA*	• *in vitro*: various cell lines • Xenograft and mouse models: BRAF-dependent melanoma, lymphoma	Pytel et al. 2016 (128) Tameire et al. 2019 (80)
		AMG-44	*NA*	• *in vitro*: various cell lines • Xenograft and mouse models: Lung cancer	Mohamed et al. 2020 (129)
		NMS-03597812	Phase I	Multiple Myeloma	NCT05027594
		HC-5404-FU	Phase I	Various solid tumors	NCT04834778
	PKR	2-aminopurine (2-AP)	*NA*	• *in vitro*: various cell lines	Weng et al. 2018 (130)
		C16	*NA*	• *in vitro*: various cell lines • Xenograft and mouse models: hepatocellular carcinoma	Watanabe et al. 2020 (131)
	P-eIF2α activity	ISRIB	*NA*	• *in vitro*: various cell lines • Xenograft and mouse models: prostate, breast, and lung cancer	Nguyen et al. 2018 (71), Jewer et al. 2020 (97), Ghaddar et al. 2021 (75)
		Trazodone	*FDA approved*	• *in vitro*: various cell lines	Harvey et al. 2019 (132)

### 5.1 Exploiting the response

Cancer therapies which activate the ISR to provoke cell death have been in development for several decades. In 1953, lymphomas transplanted into mice regressed upon intraperitoneal injection of guinea pig serum (133). In 1963, these effects were attributed to the L-asparaginase found at high concentrations within the serum (134). The first clinical trial involving L-asparaginase was completed three years later (135). Since then, several asparaginase preparations have been FDA (Food and Drug Administration) approved for treatment of pediatric and adult hematological malignancies, particularly acute lymphoblastic leukemia (136). These early therapeutic discoveries unknowingly enhanced the ISR activity: L-asparaginase converts asparagine to aspartic acid and ammonia, which triggers activation of GCN2 to drive cancer cells towards ISR-mediated apoptosis (30). The efficacy of L-asparaginase as a highly selective therapeutic agent is rooted in leukemic cells’ deficiency in asparagine synthetase. Therefore, these cancer cells are dependent on the availability of extracellular L-asparagine, which is depleted by L-asparaginase (137, 138). Importantly, normal cells synthesize enough L-asparagine to survive treatments creating an asparaginase deficiency in the TME. Additional therapies that induce nutrient stress are being created for solid tumors, including CB-839, which acts as a glutaminase inhibitor and is currently in phase II clinical trials. Depletion of intracellular glutamine by CB-839 also induces the GCN2 arm of ISR, killing glutamine-addicted tumor cells (139). While responses to CB-839 monotherapy are limited, combinatorial treatment with immunotherapeutics has demonstrated potential. Another nutrient stress activator derived from a natural quinazolinone alkaloid, halofuginone, has been shown to promote the amino acid starvation response through GCN2 activation (140, 141). This ultimately chemosensitizes esophageal and lung carcinoma cells that have high expression of NRF2 *in vitro* (109). The same study found that halofuginone had similar anti-cancer effects *in vivo* by enhancing cisplatin-mediated tumor death in an esophageal cancer xenograft model. Similarly, drugs that have shown resistance with poor efficacy in the clinic (such as Neratinib in Glioblastoma) are now being recognized for off target effects turning on adaptations through activating the ISR *via* GCN2 (110). These studies highlight the presence of redundant signaling pathways that can promote cancer cell survival and provide foundations for combinational therapy strategies targeting the ISR.

A new family of selective cancer therapies known as imipridones are showing promise in the clinic. These drugs target distinct G protein-coupled receptors (GPCRs) and the mitochondrial caseinolytic protease P (ClpP) responsible for degrading misfolded proteins. When an impridone molecule binds to ClpP, it hyperactivates its function. This induces proteolysis leading to a loss of mitochondrial function, increased oxidative phosphorylation, and the triggering of the ISR and cancer cell death (142). ONC201 (also known as TIC10) is an imipridone molecule which induces HRI- and PKR-dependent phosphorylation of eIF2α and inactivation of Akt and ERK signaling (143). These effects lead to cell death by activation of Foxo3a and TRAIL for apoptotic pathways (144). Multiple phase II clinical trials have been completed and are ongoing for the use of ONC201 in treating solid and hematological cancers (145, 146), and phase III clinical trials are currently recruiting patients with H3K27M-mutant glioma and diffuse intrinsic pontine glioma (DIPG) (NCT05580562, NCT05476939). In these gliomas, the GPCR directly antagonized by ONC201, dopamine receptor D2 (DRD2), acts to promote tumor growth (147, 148). Additionally, several *in vitro* and *in vivo* studies have demonstrated the potential of DRD2 antagonists in other cancer types characterized by DRD2 upregulation including breast, prostatic, pancreatic, blood, oral, lung, gastric, and renal malignancies (149). ONC206, an antagonist for D2-like dopamine receptors, is actively in clinical trials for the treatment of primary nervous system neoplasms (such as glioblastoma) and has shown anti-tumor success in melanoma, colorectal cancer, and endometrial cancer inducing cell death by activating the ISR (150). A therapeutic relative of ONC206, ONC212, has been shown to be effective at inducing apoptosis in acute myeloid leukemia (AML) through the induction of the ISR. The resulting ISR induction also makes B-cell leukemia 2 protein (BCL-2) inhibition therapy in AML more effective by decreasing the expression of a known resistance factor for BCL-2 inhibition, myeloid cell leukemia-1 (151). A separate study found that venetoclax, a BCL-2 inhibitor, synergizes with tedizolid, an inhibitor of mitochondrial protein synthesis, to activate the ISR causing cell death through an inhibition of glycolytic activity in venetoclax-resistant AML (152).

Domperidone, another drug that works as an antagonist of dopamine receptors, and multiple tricyclic antidepressants (TCAs) have been shown to be effective at causing colorectal cancer cell death when paired with niclosamide ethanolamine (NEN), a mitochondrial uncoupler (153). The cytotoxic effects of these drug combinations are reversed with the treatment of ISRIB, indicating that they rely on ISR signaling. In the same study, the combination therapy was extremely effective at sensitizing pancreatic ductal adenocarcinoma to standard-of-care paclitaxel treatment. PKR has been shown to be activated by the non-steroidal anti-inflammatory drug, indomethacin (INDO). Upon PKR activation by INDO in colorectal cancer, the cells become susceptible to cisplatin chemotherapy treatment. Despite these findings, more work is required to identify the direct pathway of INDO mediated PKR activation (154). Furthermore, *N,N’*-diarylureas (BtdCPU) have demonstrated the potential to activate the ISR through the HRI kinase (155). This has been demonstrated to inhibit breast cancer growth in mice carrying human breast cancer xenografts (107). Overactivation of PERK has also been a subject of interest for creating novel therapies (156). Enhanced PERK activity by the drug CCT020312 has been shown to lead to enhanced apoptosis and taxol chemosensitivity in colorectal cancer (112, 157). In addition, CCT020312 has antitumor effects in triple-negative breast cancer through cell cycle arrest, ultimately leading to apoptosis (111).

Much like activation of eIF2α kinases, inhibition of eIF2α phosphatases can extend ISR activity and promote cancer cell death. Salubrinal is a potent cell-permeable inhibitor of eIF2α phosphatases originally identified for its ability to protect against ER stress and modulate ER stress-related cell death by targeting the conserved PP1-interacting domain of phosphatase regulatory binding partners, including GADD34 and CReP (158–160). In recent years, Salubrinal and its derivatives have risen in popularity in preclinical research, both as monotherapy and in conjunction with other treatments. An *in vitro* study of hepatocellular carcinoma demonstrated a potentiation of cell death when cytotoxic agent Pterostilbene was combined with Salubrinal (124). Another study demonstrated the efficacy of Salubrinal as a treatment for inflammatory breast cancer, showing that Salubrinal increased production of ROS and reduced cell proliferation (161). In ovarian cancer, it was discovered that inhibition of the therapeutic target valosin-containing protein (VCP) enhances P-eIF2α and ATF4 expression. When these VCP inhibitors are combined with Salubrinal treatment, ATF4 expression is enhanced leading to a greater increase in cancer apoptosis than with VCP inhibition alone (162). In inflammatory breast cancer (IBC), there is a markedly high expression of genes involved with the ISR such as CCAAT enhancer-binding protein homologous protein (CHOP), PERK, and ATF4. When these IBC cells are treated with Salubrinal there is a notable increase in apoptosis and expression of ATF4 and CHOP, effects that are not observed in Salubrinal-treated control cells (161). Furthermore, Salubrinal treatment was shown to be effective at causing doxorubicin-resistant MCF-7 breast cancer cells to become more susceptible to doxorubicin-induced apoptosis through the inhibition of GADD34 (163). In complex with copper (as CoSAL), Salubrinal has been shown to promote cell death and the accumulation of DNA damage in ovarian cancer *in vitro* (164). To combat the off-target cytotoxicity of Salubrinal, the analog SAL003 was developed to show similar efficacy to its predecessor under lower concentrations (158, 165). Trastuzumab, an important chemotherapy drug in the treatment of HER2+ cancers, has been shown to have enhanced potency when paired with the analog SAL003 in resistant HER2+ gastric and breast cancer (166).

Additional eIF2α phosphatase inhibitors have been investigated in preclinical and clinical studies. Guanabenz acetate, originally marketed as an antihypertensive, has been repurposed as an anticancer drug for its inhibition of GADD34 (117, 167). Guanabenz has been shown to induce cell death in primary hepatocellular carcinoma cells and sensitize glioblastoma cells to Sunitinib treatment (115, 116). A 2015 clinical trial exploring the anti-metastatic activity of guanabenz acetate in bone cancer patients was terminated prematurely due to poor accrual (NCT024432013), and no cancer-related clinical trials for the drug are ongoing at this time. One study that showed guanabenz acetate’s ability to inhibit the growth of anaplastic thyroid carcinoma (ATC) also employed the use of Sephin1, a small molecule inhibitor of GADD34 more commonly used in neurological studies of ISR (118). Sephin1 was also able to inhibit the growth of ATC, showcasing its potential as an anti-cancer drug. However, a more recent study demonstrated that Sephin1-mediated inhibition of GADD34 may actually have protumorigenic effect by decreasing levels of antitumor immune cells such as tumor-specific T cells and a TCR+ macrophage subtype (119). The ISR is also activated *via* inhibition of CReP by Nelfinavir, first identified as a human immunodeficiency virus protease inhibitor (168, 169). Recently, Nelfinavir has been shown to inhibit proliferation in patient-derived small-cell lung cancer xenograft mouse models, both as a monotherapy and in combination with autophagy inhibitor Chloroquine (170, 171). Currently, over 20 Nelfinavir clinical trials have been completed or are ongoing, with most investigating the drug as a treatment for advanced solid tumors in combination with chemoradiotherapy.

### 5.2 Inhibiting the response

The ISR enables cells to take dynamic countermeasures when faced with extrinsic and intrinsic stressors. This plasticity is a major promoter of cell survival under adverse conditions, including cancer cell survival under genomic instability, anticancer therapy, and the stresses of the TME. Because of this role in maintaining homeostasis and promoting cell survival, the ISR may also have significant potential as a target for inhibition in the context of cancer. Specifically inhibiting the ISR may block oncogenic cellular adaptation, leading to the same outcome of cell death as seen when pushing ISR activation in the context of tumor growth.

A common method to halt the ISR is to directly inhibit the eIF2α kinases, often through an ATP-competitor. Indirubin-3’-monoxime, SP600125, and a SyK (spleen tyrosine kinase) inhibitor were all identified in a single screen as inhibitors of GCN2 kinase activity in UV-treated mouse embryonic fibroblasts (172). Since then, indirubin-3’-monoxime has been demonstrated to inhibit osteosarcoma cell proliferation and migration (120), induce paraptosis in breast cancer cells (121), and sensitize multiple myeloma cells to bortezomib-induced cell death (122). SP600125, an inhibitor of c-Jun N-terminal kinase (JNK), has shown promise in combatting oral squamous carcinoma, lung adenocarcinoma, cholangiocarcinoma, colon carcinoma, pancreatic cancer, glioblastoma, and doxorubicin-resistant stomach cancer (123). SyK is primarily expressed in hematopoietic cells and is a key component of the B-cell receptor signaling pathway crucial for B cell survival and for antigen-mediated activation, proliferation, and differentiation. Several SyK inhibitors have been evaluated in clinical trials (173). Notably, none of these GCN2 inhibitors act exclusively on GCN2.

The ATP competitive GCN2 inhibitor, GCN2iB, reduces the ISR and prevents GCN2 activation upon nutrient limitations (174). This novel inhibitor demonstrates that inhibition of GCN2 sensitizes cancer cells with low basal-level expression of asparagine synthetase to other agents such as the antileukemic agent L-asparaginase in acute lymphocytic leukemia. Preclinical models of hepatocellular carcinoma confirm that with combined dietary arginine deprivation and senotherapy, GCN2 inhibition promotes tumor regression (88). Growth inhibition was also evident using GCN2iB in cell line-derived and patient-derived xenograft models of prostate cancer (76). GZD824, a multikinase inhibitor also known as Olverembatinib, has been shown to halt the GCN2 pathway in human fibrosarcoma and non-small cell lung cancer *in vitro* (175). While its ability to inhibit an array of kinases may allow GZD824 considerable potential to induce off-target effects, several clinical trials are currently active or recruiting patients with hematological cancers, including one phase III clinical trial for patients with tumors that are resistant to at least two second-generation tyrosine kinase inhibitors (NCT05311943). Initially, GZD824 was identified as an inhibitor of BCR-ABL, the fusion protein with constitutively active tyrosine kinase activity, commonly associated with chronic myeloid leukemia (176). Interestingly, BCR-ABL inhibitors have been shown to prevent the activation of both GCN2 and PERK, thereby inhibiting downstream ATF4 induction in chronic myeloid leukemia (177).

Direct PERK inhibitors include GSK2606414, GSK2656157, LY-4, and AMG-44 (127, 178, 179). Unfortunately, previous research has shown off-target toxicity of PERK inhibition, particularly in pancreatic cancer. This toxicity is mediated by type 1 interferon signaling, and neutralization of this signaling has been shown to protect the healthy pancreatic tissue against PERK-inhibitors (180). Currently, a Phase I clinical trial for PERK inhibitor NMS-03597812 is recruiting patients with relapsed or refractory multiple myeloma (NCT05027594). Also recruiting, is a Phase Ia clinical trial for HC-5404-FU, another PERK inhibitor, seeking patients with renal cell carcinoma, gastric cancer, metastatic breast cancer, small cell lung cancer, and other solid tumors (except rapidly progressing neoplasms, such as pancreatic cancer) (NCT04834778).

A widely used inhibitor for PKR is 2-aminopurine (2-AP) an analog of guanosine and adenosine typically used at millimolar concentrations (181). This has been seen to affect EMT in lung cancer cells by suppressing TGF-β signaling though the influence of PKR here remains unknown and being used at such high concentrations increases likely hood of targeting several other kinases (130). An alternative PKR inhibitor is the small molecule C16, an oxindole/imidazole derivative (182). Although primarily studied in neurological contexts, C16 has shown to suppress proliferation in hepatocellular carcinoma cell lines and xenograft mouse models partially by decreasing angiogenesis (131). This raises the idea that targeting angiogenesis *via* PKR might be useful in other cancer subtypes, though future studies should consider its potential to inhibit immunotherapies. Similar to PKR, HRI has few known inhibitors. Aminopyrazolindane was identified in an HRI-kinase assay to act as selective inhibitor (183, 184). Sadly, *in vivo* the drug was cleared quickly and showed limited bioavailability for future studies. While few known inhibitors are currently in development for HRI compared to other eIF2α kinases, HRI inhibitors like aminopyrazolindane could have specific potential to combat the responses triggered by oxidative stress and dysregulation of iron homeostasis that may otherwise contribute to anemia. Anemia is remarkably common in cancer patients and while the numbers vary widely by cancer type and disease stage (as well as variation in designation of what constitutes “low” hemoglobin), studies have generally found 30-90% of cancer patients to be anemic (185). Moreover, chemotherapy is known to induce anemia. This highlights the potential for combinatorial therapies that utilize HRI inhibitors to combat oxidative stress.

As highlighted throughout, ISRIB is one of the ISR inhibitors that works downstream of all four eIF2α kinases by binding to eIF2B. This binding triggers an inhibitory allosteric change at the P-eIF2α binding site of eIF2B, thereby allowing free eIF2α to bind and promote the ternary complex to relieve ISR inhibition (186, 187). Interestingly, ISRIB functions as an effective modulator of P-eIF2α-mediated responses, but it does so without the pancreatic toxicity of other ISR inhibitors (such as PERK inhibitor GSK2606414) (188). Combinatorial treatment with ISRIB and imatinib both attenuated resistance-driving signaling pathways and more effectively eradicated chronic myeloid leukemia cells— *in vitro* and *in vivo*— than either drug alone (189). In combination with bortezomib, ISRIB has been shown to both protect bortezomib-sensitive multiple myeloma cells against apoptosis and induce paraptosis in bortezomib-insensitive breast cancer cells (190). ISRIB will likely not be available for clinical use due to poor solubility. Nevertheless, new analogs are in development for improved potency and solubility (*e.g.* 2BAct) (191). Direct prevention of P-eIF2α mediated reductions in ternary complex formation may also be a novel way to inhibit the ISR. Trazodone, an FDA-approved antidepressant, was shown to have similar effects as ISRIB working in this manner, yet the exact mechanism of regulation remains to be defined (132, 192). Altogether, the exponential success in basic research supports the idea that ISR-targeting therapeutics may be particularly useful in combatting an array of diseases, including highly resistant tumors (193).

## 6 Conclusions

The ISR is evolutionarily conserved and essential for normal mammalian development (194–196). Mutations in ISR factors have been associated with developmental malformation, as well as, cognitive, metabolic, and immune dysfunction (193, 197). The consensus is that the ISR plays a pivotal role as a molecular rheostat to fine-tune cellular adaptation mediated by translational reprogramming. As a central regulator, the ISR can be a favorable target to counter various pathologies depending on the disease context.

A wealth of research has presented the ISR as an oncogenic stress-induced translational program which enables swift cell state transitions to facilitate tumor growth, metastasis, and resistance. There are numerous mechanisms by which the ISR dynamically modulates translation— ranging from the recruitment of alternative initiation factors, selective translation of distinct transcripts, to utilization of noncanonical start codons. While the ISR operates as a means by which normal cells can adapt rapidly to non-oncogenic stressors (misfolded proteins, oxidative stress, heme-imbalance, etc.), it also exemplifies the capacity of cancer cells to hijack the pathways, networks, and fail-safes of their healthy precursors. This exploitation of typical cell functionality serves to promote neoplastic transformation. From the earliest stages of a precancerous lesion to the latest stages of metastatic disease, the ISR plays a crucial role in enabling tumors to handle the various oncogenic insults produced by their own development. Outside these intrinsic stressors, clinically available therapeutics provide extrinsic stimuli leading to the activation of the ISR. As a result, ISR-induced cell plasticity can become a driving force for therapeutic resistance with a need for pharmacological remedy.

The development of highly plastic, drug-resistant tumors by activation of the ISR has proven to be a formidable barrier to cancer therapy. On the other hand, a tumor’s overreliance on the ISR presents the translational program as a therapeutic liability. Both agonists and antagonists of the ISR have shown promise in promoting cancer cell death when combined with existing therapies. As such, there is an incentive for the development of novel ISR-modulating therapies capable of preventing ISR-mediated adaptations. Since therapeutic resistance is a major functional output of the ISR in cancer, there is more work to be done in determining the optimal partners for ISR modulators in combinatorial treatment approaches.

As outlined in this review, there are several eIF2α kinase inhibitors actively enrolled in clinical trials for a variety of cancer types. It is imperative to consider and investigate the potential unintended effects of these (often nonspecific) inhibitors to ensure that any possible off-target effects are rigorously explored to prevent toxicity, similar to what has been observed with PERK inhibition in pancreatic cancer (198). In service of the same goal, developing analogs of current therapeutics with more specific activity may increase drug efficacy while decreasing effective concentrations; this may also reduce off-target cytotoxicity. It will be useful to limit the breadth of ISR-modulating therapies by targeting specific pathways further downstream of eIF2α phosphorylation to avoid the broader impact of upstream kinase inhibition and redundancy between kinases. The potential of small molecules like ISRIB have shown promise in mouse models without off target toxicity, though the bioavailability still needs to be improved (199). Further identification and characterization of downstream interactors will aid in this endeavor. Repurposing FDA approved drugs, such as Trazodone for inhibiting ISR activity, may also be fruitful in future clinical trials.

Another area of focus will be to elucidate under what circumstances therapeutics should aim to activate or inhibit the ISR in cancer. In certain instances, extended activation of the ISR overwhelms basic cellular functions, resulting in a push towards cell death. However, activation of the ISR can enhance cancer cells’ plasticity and prevent cell death under therapeutic stress. Because of this dual nature of the ISR, a prominent dilemma is determining the specific circumstances under which upregulation of the ISR is more beneficial than downregulation (and vice versa). This will be key for future therapies to control pro-death or pro-survival mechanisms through ISR signaling. There is value in further investigating the variable role of the ISR in different cancer types and subtypes, tumor stages, and beyond cancer– in exploring ISR modulating treatments for metabolic and age-related disorders. Therapeutically fine-tuning ISR signaling is a formidable approach to overcome environmental barriers and therapy resistance to provide improved cancer targeting strategies to the clinic.

## Author contributions

The outline was organized by CSC. All authors contributed to the article and approved the submitted version. 
